# 靶向CD19体内CAR-T细胞治疗高危难治性急性B淋巴细胞白血病1例并文献复习

**DOI:** 10.3760/cma.j.cn121090-20260510-00234

**Published:** 2026-08

**Authors:** 玉 李, 飞昀 马, 丽华 张, 婧 任, 三斌 王

**Affiliations:** 中国人民解放军联勤保障部队第九二O医院血液内科，昆明 650032 Department of hematology, 920th Hospital of Joint Logistics Support Force of People's Liberation Army of China, Kunming 650032, China

**Keywords:** 白血病，B细胞, 难治性, 免疫疗法, 抗原，CD19, Leukemia, B cell, Refractory, Immunotherapy, Antigen, CD19

## Abstract

**目的:**

探讨一款新型慢病毒载体介导的靶向CD19体内嵌合抗原受体（CAR-T）细胞治疗在1例多线难治性急性B淋巴细胞白血病（B-ALL）患者中的安全性、药代动力学特征及临床疗效。

**方法:**

1例于联勤保障部队第九二O医院就诊的B-ALL患者接受单次静脉输注慢病毒载体介导的靶向CD19体内CAR-T细胞治疗，剂量为7×10^8^ TU，收集和分析相关安全性、药代动力学（PK）与疾病评估等数据；并对现有体内CAR-T细胞治疗临床数据进行复习。

**结果:**

患者为28岁男性，患有高危型多线难治性B-ALL，输注后第16天达到微小残留病阴性完全缓解，并持续至第50天，后行造血干细胞移植。不良反应主要表现为输注后24 h内的急性炎症反应以及CAR-T细胞扩增期的细胞因子释放综合征。药代动力学监测显示，外周血CAR-T细胞于输注后第6天即被检出，第14天达到峰值（8 629.38个细胞/µl）。文献查阅显示体内CAR-T细胞治疗临床研究尚处于早期探索，相关临床证据有限，且在此前国内外均无该技术在B-ALL患者中研究数据的相关报道。

**结论:**

患者所用靶向CD19体内CAR-T细胞具有可控的安全性和较强的扩增能力，能够快速诱导缓解，从而为患者创造宝贵的移植窗口。本例初步证实了体内CAR-T细胞治疗用于B-ALL高危多线难治人群的可行性和潜在临床价值，为临床应用提供了重要的实践参考。

## Abstract (en)

**Objective:**

To evaluate the safety, pharmacokinetic profile, and preliminary efficacy of a novel lentiviral-based CD19-targeted in vivo chimeric antigen receptor T（CAR-T）cell therapy in a patient with multiply relapsed/refractory B-acute lymphoblastic leukemia（B-ALL）.

**Methods:**

A 28-year-old patient with B-ALL at the 920th Hospital of Joint Logistics Support Force, received a single intravenous infusion of a lentiviral-based CD19-targeted in vivo CAR-T product at a dose of 7×10^8^ TU. The safety and pharmacokinetic profiles and disease response data were collected and analyzed. Additionally, the outcomes of the current clinical studies on vivo CAR-T cell therapy were reviewed.

**Results:**

The patient with high-risk and heavily pretreated B-ALL achieved minimal residual disease negative complete remission by day 16 post infusion, which was sustained until day 50 and enabled subsequent hematopoietic stem cell transplantation. The adverse events mainly consisted of an acute inflammatory reaction within 24 hours after infusion and cytokine release syndrome during the CAR-T expansion phase. Pharmacokinetic monitoring detected peripheral blood CAR-T cells on day 6, with a peak level of 8 629.38 cells/µl on day 14. Literature review suggested that clinical research on in vivo CAR-T cell therapy remains at an early stage, with limited evidence available, and that no prior clinical outcomes of its application in B-ALL patients were reported.

**Conclusion:**

CD19-targeted in vivo CAR-T cell therapy demonstrated manageable safety and robust in vivo expansion in our patient, enabling rapid remission and creating a critical window for transplantation. Our case preliminarily supports the feasibility and potential clinical value of in vivo CAR-T cell therapy in high-risk, heavily pretreated B-ALL patients and provides a valuable practical reference for clinical application.

复发难治性急性B淋巴细胞白血病（R/R B-ALL）的治疗仍是临床面临的严峻挑战。尽管靶向CD19的嵌合抗原受体T（CAR-T）细胞治疗已取得突破，但其临床应用仍受限于高昂成本、漫长制备周期及多线治疗后患者T细胞功能耗竭所带来的制备失败风险。基于病毒或非病毒载体递送的体内CAR-T技术应运而生，旨在通过静脉输注包裹CAR基因的载体直接在患者体内生成CAR-T细胞，从而克服传统体外制备的局限，为多线难治患者提供了新的治疗可能。然而，目前该领域临床数据有限，尤其是针对B-ALL的临床应用还未有文献报道[1]。本文详细阐述1例高危多线难治性B-ALL患者接受慢病毒载体介导的靶向CD19体内CAR-T细胞治疗的全程管理、药代动力学（PK）、药效动力学（PD）特征和不良反应及应对策略，以期为深入理解此类新型疗法的临床特性、潜在优势与挑战提供参考。

## 病例资料

患者，男性，28岁，2025年5月因间断胸背部疼痛伴呼吸困难、发热入院治疗。完善相关检查，骨髓细胞形态学示原始幼稚淋巴细胞占82％，流式细胞术免疫分型提示异常原始幼稚B淋巴细胞（CD38^+^、DR^+^、CD19^+^、CD22^+^）占53.54％。基因筛查结果提示TCF3::PBX1融合基因阳性，TP53突变阳性（变异等位基因频率52.33％，变异位点c.632C>T,p.T211I），染色体核型为45~46,XY,der（9）t（9;17）（p13;q21）,−17,der（19）t（1;19）（q23;p13），诊断为B-ALL。

2025年5月28日起予以VDLP方案（长春新碱2 mg，第1天；柔红霉素50 mg，第1、15天；门冬酰胺酶3 180 IU，第8天；泼尼松50 mg第1～7天，60 mg第8～21天，第22天后逐渐减停）诱导治疗，15 d后疗效评估为完全缓解。6月10日、6月17日行鞘内注射（甲氨蝶呤15 mg+阿糖胞苷50 mg+地塞米松5 mg）预防中枢神经系统白血病。7月2日骨髓形态学检查无异常，患者接受CAM-L方案（环磷酰胺1 200 mg，第1天；阿糖胞苷130 mg第3～6天、第10～13天；6-巯基嘌呤100 mg，第1～14天；门冬酰胺酶3 320 IU，第2天）巩固治疗并行腰椎穿刺和鞘内注射1次。8月9日患者再次接受巩固化疗，期间并行鞘内注射2次，骨髓细胞形态学评估维持完全缓解。此后患者未规律化疗。9月21日复查，骨髓细胞形态学示原始幼稚淋巴细胞占10.5％，提示复发。予维奈克拉（400 mg，第1～7天）联合VDP方案（长春新碱2 mg，第1、8、15天；柔红霉素50 mg，第1天；泼尼松50 mg第1～7天，60 mg第8～21天，第22天后逐渐减停）治疗，并于9月28日、10月5日行腰椎穿刺和鞘内注射。10月15日，骨髓原始幼稚淋巴细胞占比升至28％，外周血可见2％的原始幼稚淋巴细胞，提示疾病进展。遂改用VDLP方案，化疗14 d后复查骨髓原始幼稚淋巴细胞比例达49％，外周血原始幼稚淋巴细胞占4％，治疗无效。

11月5日予地西他滨（33 mg，第1～5天）和维奈克拉（400 mg，第2～12天）进行挽救治疗，仍疗效不佳。11月19日，患者骨髓原始幼稚淋巴细胞比例升至81％，疾病进展迅速，流式细胞术检测示CD22阳性，于11月22日至29日予奥加伊妥珠单抗（1 mg，第1、8天）、维奈克拉（400 mg，第1～8天）及地塞米松（5 mg第1～2天、10 mg第3天、15 mg第4～8天）治疗，一周后骨髓原始幼稚淋巴细胞比例降至17.5％，TCF3::PBX1检测（荧光定量PCR）提示融合基因比率为94.97％。综合考虑患者为多线难治、预后极差的化疗耐药病例，现有治疗手段疗效甚微，因此建议患者尝试新型体内CAR-T细胞治疗。

2025年11月28日，主治医师与患者及家属进行知情谈话，医师详细告知临床试验的背景、目的、步骤、预期持续时间、试验药物详细信息、预期获益及不能获益的可能性、可能的风险、发生损害时的补偿与治疗等情况；给予患者及家属充分的时间考虑。患者及家属表示已理解本试验的情况，自愿同意参与临床研究。患者于2025年11月28日10时56分签署知情同意书，研究者于10时57分完成签署知情同意书，一式两份，一份已交给受试者保存，一份保存于研究中心，本研究已通过中国人民解放军联勤保障部队第九二〇医院伦理委员会审批（批件号：伦审2023-1）。

2025年11月29日，输注前1天（D-1），患者基线血常规示WBC 0.88×10^9^/L、ANC 0.51×10^9^/L、绝对淋巴细胞计数（ALC）0.36×10^9^/L、PLT 65×10^9^/L，吸氧流量为3 L/min。

2025年11月30日，输注当天（D0）患者一般状况平稳。体内CAR-T输注前30～40 min（11时）预防性予地塞米松20 mg、异丙嗪20 mg、甘露醇25 mg，随后11时40分左右以0.5～1 ml/min输注总剂量7×10^8^ TU的慢病毒载体颗粒，于12时28分完成输注，过程顺利。慢病毒载体颗粒由上海锋寻生物科技有限公司自主研发和制备，该患者所输注剂量由临床前动物实验安全剂量推导，并结合同类产品临床探索剂量得出。输注结束后约2.5 h，患者出现低血压91/54 mmHg（1 mmHg＝0.133 kPa），心率142次/min，立即予调整氧流量至15 L/min，并先后于15时予托珠单抗400 mg，15时30分予地塞米松10 mg，16时开始予去甲肾上腺素18 mg及甲泼尼龙40 mg等处理后，血压于6 h内逐步回升并趋于稳定。输注后约4 h患者出现膝盖、腰部等阵发性疼痛，予止痛药后减轻，随后出现双手、嘴唇发麻。细胞因子检测显示此时白细胞介素（IL）-6、IL-8、IL-10、γ干扰素（IFN-γ）和肿瘤坏死因子（TNF）-α等达到峰值，IL-6 42 608.05 pg/ml、IL-8 44 210.00 pg/ml，输注后12 h迅速回落。输注后约4.5 h，当日最高体温38°C，约30 min后降至正常。当晚输注后约8.5 h血压再次波动下降，21时左右予第2剂托珠单抗400 mg处理后改善。输注后约12 h双臂持续疼痛，24时（0时）50分予第2剂地塞米松10 mg。

输注后第1天（D1）患者生命体征趋于平稳，间断双臂疼痛，氧流量降至5 L/min，21时10分予以第3剂地塞米松10 mg。实验室检查：ALC显著下降至0.04×10^9^/L，PLT降至34×10^9^/L；肝功能：丙氨酸氨基转移酶（ALT）441 U/L，天门冬氨酸氨基转移酶（AST）545 U/L，γ-谷氨酰转移酶（γ-GGT）733 U/L；炎性相关指标：铁蛋白53 130 ng/ml、C-反应蛋白（CRP）109.3 mg/L。多个细胞因子水平下降：IL-6 1 331.09 pg/ml、IL-8 2 588.85 pg/ml。

D2患者生命体征平稳。去甲肾上腺素逐渐减量并于D3停用。实验室检查显示ALC逐步回升，PLT仍有下降趋势，肝功能指标下降，D-二聚体于D2达到最高值40.51 mg/L，纤维蛋白原降解产物达到最高值113.5 mg/L，炎症指标铁蛋白、CRP及细胞因子水平显著回落并接近基线。D5患者左侧鼻腔出血，D6至D12诉头痛。输注后第2周相继出现腰背部疼痛、颈部双侧淋巴结肿痛、头面部麻木、构音障碍、心动过速、乏力、恶心等症状，无发热，于D8、D10分别予托珠单抗400 mg后，症状有所缓解并逐渐恢复。该时段内实验室检查变化如下：ALC持续恢复，D11起显著上升，在D14达峰值（13.58×10^9^/L）；PLT于D7降至14×10^9^/L后逐步回升；AST、铁蛋白于D12轻度上升后下降；IL-6自D6起处于稍高水平（1 632.76～1 964.97 pg/ml），持续至D12。D28血常规检查示ANC 0.42×10^9^/L，PLT恢复至85×10^9^/L，ALC恢复正常。输注后相关实验室检查见附图1、2（**扫描本文首页二维码查阅**）。

PK监测显示，外周血CAR-T细胞于D6首次检出，D14达峰值8 629.38个细胞/µl，此时CAR阳性细胞比率为81.95％；至D28，CAR-T细胞浓度为269.06个细胞/µl，CAR阳性细胞比率为25.69％。CAR-T细胞浓度和阳性率变化情况见图1。

**图1:**
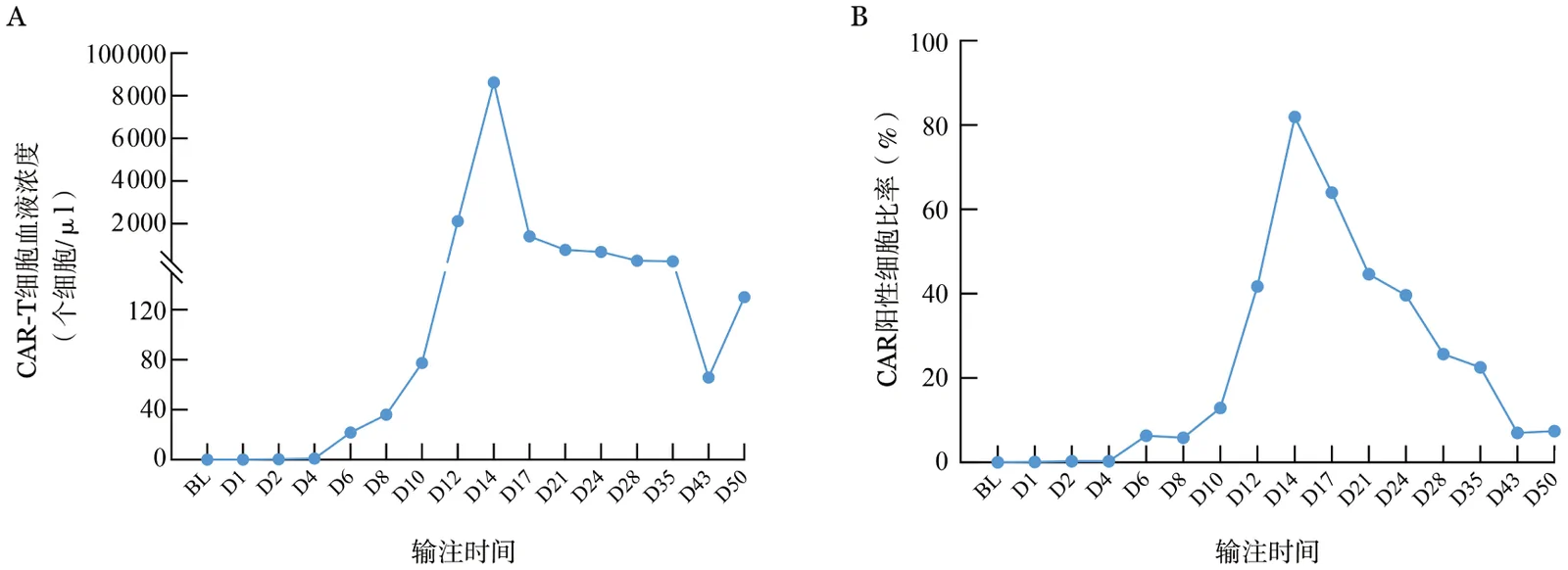
高危难治性急性B淋巴细胞白血病患者接受靶向CD19体内嵌合抗原受体T（CAR-T）输注后CAR-T细胞动力学变化 **A** 外周血CAR-T细胞浓度随时间的变化情况；**B** 外周血CAR阳性细胞比率随时间的变化情况 **注** BL：基线水平

D9骨髓细胞形态学示原始幼稚淋巴细胞占2.5％，流式细胞术检测见3.66％的异常B淋巴细胞，骨髓形态学完全缓解；D16骨髓形态学及流式细胞术检测均未见异常细胞，TCF3::PBX1融合基因比例检测为0，提示微小残留病（MRD，本文中MRD检测均指流式细胞术检测，灵敏度为10⁻⁴）阴性缓解；D28骨髓检查仍维持缓解状态。后期随访中，D35检测到TCF3::PBX1融合基因比例为99.97％，提示早期分子学复发可能；D50骨髓流式细胞术检出1.7％的异常B淋巴细胞，提示MRD转阳，考虑患者有疾病复发风险，2026年2月6日行半相合异基因造血干细胞移植挽救治疗，供者为其父，移植后第13天实现粒细胞植入，第15天血小板植入。移植后第19天骨髓流式细胞术和基因检测显示MRD阴性，TCF3::PBX1融合基因及TP53基因突变均为阴性，首次骨髓T细胞嵌合状态分析显示嵌合体为完全供者型。患者移植后未出现移植物抗宿主病。与未接受体内CAR-T细胞治疗的移植患者相比，生命体征与实验室检查未见显著异常。患者于2026年3月29日（移植后第51天）进行检查，结果提示TCF3::PBX1融合基因及TP53基因突变仍为阴性，后患者失访。

## 讨论及文献复习

体内CAR-T（in vivo CAR-T）技术通过载体递送CAR基因到患者体内T细胞，改造T细胞形成具有肿瘤杀伤活性的CAR-T细胞，无需体外采集、扩增和培养，极大地降低了传统CAR-T细胞治疗的制造复杂度，缩短了患者的等待时间，减少了细胞治疗药物的生产成本。

体内CAR-T技术的基础研究主要围绕载体的开发和优化展开，得益于病毒学、RNA疗法以及纳米医学的进展，目前该技术主要有病毒与非病毒两大类载体[2]。病毒载体（以慢病毒载体和腺相关病毒载体为代表）转导效率更优，病毒包膜或衣壳蛋白具有明确的细胞嗜性与可遗传改造特性，能够实现对特定T细胞亚群的精准靶向。病毒载体具有相对成熟的生产体系，可实现高滴度、符合临床级标准的大规模制备，并在长期临床应用中积累了充分的安全性数据[1]。非病毒载体（主要包括聚合物纳米颗粒、脂质纳米颗粒等）为体内CAR-T工程提供了一种替代策略，其核心优势在于更好的安全性，规避了病毒载体潜在的免疫原性与插入突变风险，具有良好的生物相容性与可降解性，其递送基因可瞬时表达等。在制备方面，非病毒载体无需依赖细胞底物及复杂的纯化流程，工艺更为简洁，便于实现规模化生产，同时有效降低批次差异与生产成本[2]。简言之，病毒载体更高效，非病毒载体安全性更好。

近年来，体内CAR-T技术虽已取得快速进展并进入临床探索阶段，但仍面临诸多挑战，主要包括体内转导效率较低、体内生成CAR-T细胞存续时间短、不可控扩增或迟发再扩增等。体内递送需克服天然生物学屏障；病毒载体可因预存免疫（如针对腺相关病毒的中和抗体）被清除，降低转导效率；载体还要面临补体介导的灭活以及巨噬细胞的吞噬清除，这进一步限制了有效转导[1]。临床数据显示，尽管体内CAR-T细胞的扩增动力学能达到与体外CAR-T细胞相当水平，但体内生成的CAR-T细胞的持久性还有待进一步验证[3]。整合性病毒载体的技术也可能出现不可控的长期持久性或迟发的再扩增，延长可导致在靶毒性（如B细胞发育不全）或迟发性炎症事件等发生[4]。

本例患者所使用体内CAR-T产品基于慢病毒载体递送平台，通过多重设计优化以应对上述挑战。首先经过VSV-G囊膜蛋白改造降低病毒载体泛嗜性与免疫原性，同时于囊膜表面过表达CD47并敲除MHCⅠ分子以降低载体的吞噬清除风险，从而提高转导成功率。其次，采用特异性双信号识别机制（CD3和T细胞激活共刺激分子）实现精准的T细胞靶向和静息T细胞活化，以改善体内CAR-T细胞动力学特征。另外，该产品引入分子开关设计，在临床需要时可借助靶向蛋白降解技术快速清除细胞表面CAR分子，从而终止CAR‑T细胞的增殖，增强治疗过程的可控性。

第1个体内CAR-T细胞治疗的临床研究开始于2024年[4]，相关研究目前仍处于早期临床探索阶段，临床证据相对缺乏，研究队列通常较小，适应证也不尽相同。截至2026年6月，有文献或会议发表临床数据的体内CAR-T产品包括深圳虹信生物科技有限公司研发的HN2301、比利时生物技术公司EsoBiotech研发的ESO-T01、美国生物技术公司Kelonia Therapeutics研发的KLN-1010。HN2301的临床研究显示，5例多线难治性系统性红斑狼疮（SLE）患者接受脂质纳米颗粒递送mRNA（LNP-mRNA）体内CAR‑T细胞治疗后，临床症状及血清学指标均获改善，5例患者SLEDAI‑2K评分均下降，给药后B细胞被迅速清除，但1到2个月后有回升迹象。安全性方面，未发生≥3级细胞因子释放综合征（CRS）或免疫细胞相关神经毒性综合征（ICANS），仅见低级别CRS及一过性淋巴细胞减少，证实该平台在自身免疫病中具有良好的短期安全性及快速药效动力学效应[5]。ESO-T01的临床试验研究人群为复发难治性多发性骨髓瘤（RRMM）患者，首次报道的4例患者客观缓解率（ORR）为100％，其中2例达严格意义的完全缓解（sCR）；安全性方面，所有患者均发生CRS（75％患者发生3级），另有1例出现1级ICANS，不良反应经对症治疗后均得到有效控制[6]。2026年4月，该产品另一项临床研究结果显示，5例RRMM患者中有4例获得客观缓解，3例患者实现sCR；所有患者均发生了≥3级的不良反应，4例患者发生CRS，1例患者发生ICANS，研究中观察到短暂的血细胞减少；提示ESO-T01具备在临床使用的可行性但也具有较高的不良反应发生率[3]。KLN‑1010更新的Ⅰ期临床研究目前入组18例患者，14例RRMM患者完成首次疗效评估，输注后1个月均达MRD阴性缓解，ORR为100％，且缓解程度随着时间进一步加深，其中随访超过6个月的4例患者中3例患者达到sCR；安全性方面，15例患者发生1～2级CRS，无3级CRS发生（本研究中将CRS与输注后短期内的输注反应区分报告），2例患者观察到ICANS，其中1例为3级[7]。未预防用药的5例患者中3例出现输注相关反应（1例为3级，判定为剂量限制性不良反应），使用预防用药的13例患者中未观察到输注反应，常见不良反应还包括短时间血细胞减少；PK数据显示其血液中浓度峰值（Cmax）水平与已获批产品Ciltacabtagene autoleucel相当[7]–[8]。

截至2026年6月，尚无体内CAR-T细胞治疗在B-ALL中的临床数据的文献或会议摘要发表。本文详细描述1例B-ALL患者接受慢病毒载体介导的靶向CD19体内CAR-T细胞治疗过程，旨在为临床深入理解体内CAR-T产品特性及其实践应用提供参考。

本例患者属于高危型B-ALL，既往经历5线标准治疗仍难治。首次复发发生于第2次巩固治疗后2个月内，复发后对多线化疗耐药，疾病进展快速，末次治疗接受基于奥加伊妥珠单抗的方案，虽部分降低肿瘤负荷，但仍未达完全缓解，属于典型多线难治病例，后续治疗选择有限。抗体类药物及体外CAR-T细胞治疗费用高昂，体外CAR-T细胞治疗还需等待较长制备周期，且多线治疗后患者T细胞功能可能受损，影响体外CAR-T细胞制备的成功率，而体内CAR-T细胞治疗为此类患者提供了新的潜在治疗路径。本例患者在疾病再次进展前及时接受了体内CAR-T输注且无需清淋处理，后续监测显示，输注后2周左右患者实现了MRD阴性完全缓解，并持续至输注后约50 d，为患者争取到宝贵的移植窗口期。

输注后，CAR-T细胞最早于D6在外周血中被检出，与同类在研产品ESO-T01相近[5]。CAR-T细胞扩增的Cmax为8 629.38个细胞/µl，高于ESO-T01的峰值2 601个细胞/µl[5]，峰值阳性率为81.95％，与KLN-1010研究中的最高阳性率相当。本例患者的不良反应集中表现在输注后24 h内以及CAR-T细胞扩增期。输注后24 h内主要表现为急性炎症反应以及生化指标异常（ALC骤降，CRP和铁蛋白显著升高等），经过激素、托珠单抗以及对症治疗后快速缓解并恢复至基线水平。此类急性炎症反应为体内CAR-T细胞治疗相较于体外CAR-T细胞治疗特有的不良反应，可能与病毒载体或纳米脂质体颗粒入血后激活先天性免疫系统、引起细胞因子在体内快速释放有关，也可能与T细胞激活介导的促炎因子释放相关。该反应是否归属于输注反应，或是CRS，或是独立定义尚待进一步讨论界定。可通过预防性使用糖皮质激素或托珠单抗来减轻该反应程度[3],[6]–[7]，未来也可研究和尝试联合用药（如激素、托珠单抗或芦可替尼）的预防策略进一步减轻，但联合用药对载体转导效率以及T细胞功能的影响还需评估。

此外，在其他研究和本例患者中均观察到D1出现短暂的外周血T细胞计数急剧下降，未经干预便可自行恢复，该现象也为体内CAR-T细胞治疗所特有，An等[3]认为可能由T细胞激活后从外周血中向外迁移导致。Xu等[6]在新发表的研究中对该现象的机制进一步讨论，认为与载体介导的先天免疫激活有关，其发生的可能机制包括细胞因子驱动的细胞再分布、血管渗透性的改变、暂时性的造血抑制以及由固有免疫感应通路引起的T细胞隔离等。针对该现象的确切机制以及其对治疗结果的影响尚需进一步研究确认。CAR-T细胞扩增期间，患者主要表现为CRS相关症状，包括头痛、腰痛、颈部双侧淋巴结肿大等，予以托珠单抗治疗后缓解。该阶段PK检测显示CAR-T细胞在体内快速增殖、细胞因子检测显示IL-6持续较高水平，提示这些相关症状与CAR-T细胞在体内扩增有关，与传统体外CAR-T细胞产品的毒性特征相似。

本研究作为一项探索性个案观察，存在局限性。首先，单中心单例观察仅能获得初步结论，其缓解率和安全性数据均需在更大规模临床研究中验证。其次，随访期仅至移植后51天，且在检测到疾病复发信号后便停止了对CAR-T细胞的检测，未能充分评估体内CAR-T细胞的长期存续性及迟发性毒性。此外，受限于病例数量及研究设计的缺陷，本研究未能在检测到复发信号后进行肿瘤细胞的相关检测，限制了对复发机制的分子层面探究。我们正在积累更多病例，并计划在后续研究中纳入更多机制探索性指标，以期为该技术的临床转化提供更坚实的证据基础。

综上所述，体内CAR-T技术正处于从概念验证向临床应用转化的关键阶段。目前本病例为国内首例发表的体内CAR-T技术在B-ALL领域的临床应用与尝试。患者所用靶向CD19体内CAR-T细胞治疗具有可控的安全性和较强的扩增能力，能够快速诱导缓解创造移植窗口，初步证实了该技术用于B-ALL高危多线难治人群治疗的可行性和初步临床价值，为临床应用提供了重要的实践参考。

## Supplementary Material


